# Our Contributors

**Published:** 1866-05

**Authors:** 


*Our Contributors.*—Some of our promised contributions are being sent in. We are in receipt of an article from our esteemed friend, Dr. V. H. Taliaferro, of Columbus, Georgia, but too late for this issue. It will appear in the June number. Enthusiasm and sound sense, in ante-war-times, characterized the many articles furnished this journal from his pen. The hardships and privations of camp life for four years, have not, we are glad to see, destroyed his interest in, nor his knowledge of, those great principles in medical science which he so successfully studied in years gone by. His productions are always welcome to our pages. Would that all men were as true to the principles of honor and justice as he, in whatever sphere he moves—whether in the tented field, as commander of a regiment, in the sick room as prescribing physician, or in the study, seeking the hidden truths of medical science.

